# Targeting the Orexin System for Prescription Opioid Use Disorder

**DOI:** 10.3390/brainsci10040226

**Published:** 2020-04-10

**Authors:** Alessandra Matzeu, Rémi Martin-Fardon

**Affiliations:** Department of Molecular Medicine, The Scripps Research Institute, La Jolla, CA 92037, USA; rmartinf@scripps.edu

**Keywords:** orexin, prescription opioid use disorder, oxycodone, remifentanil, fentanyl

## Abstract

Prescription opioids are potent analgesics that are used for clinical pain management. However, the nonmedical use of these medications has emerged as a major concern because of dramatic increases in abuse and overdose. Therefore, effective strategies to prevent prescription opioid use disorder are urgently needed. The orexin system has been implicated in the regulation of motivation, arousal, and stress, making this system a promising target for the treatment of substance use disorder. This review discusses recent preclinical studies that suggest that orexin receptor blockade could be beneficial for the treatment of prescription opioid use disorder.

## 1. Introduction

The abuse of opioids and opioid overdoses have escalated dramatically in recent years. In 2017, around 1.7 million people in the United States suffered from substance use disorders that are related to prescription opioid pain relievers [1]. Major obstacles to the treatment of opioid use disorder (OUD) are successful detoxification and the prevention of overdose and relapse [2]. Pain is clinically managed using various potent analgesics, such as oxycodone, hydrocodone, fentanyl, and remifentanil. The nonmedical use of these prescription opioids has become a major concern because of dramatic increases in abuse and overdose [3,4,5]. Only a few medications are available for the treatment of OUD. Methadone and buprenorphine, for example, are opioid receptor agonists and are commonly used as replacement therapy for the treatment of OUD. These medications can be helpful to prevent opioid craving and relapse. However, they are poorly utilized because of their restricted access, the need to be administered in a maintenance regimen, the risk of overdose, and the social stigma associated with OUD [1]. This indicates that OUD still requires significant medical attention, thus underscoring the need to discover novel pharmacotherapies. 

The accumulating evidence indicates that the orexin (Orx) system may be a treatment target for substance use disorder. Orexin projections are found throughout the brain [6,7] and participate in the regulation of several physiological functions (e.g., the regulation of feeding, sleep/wake states, and energy homeostasis; for a review, see [8]). Orexin neurons play a role in modulating reward function, particularly drug-directed behavior [9]. It has been shown that Orx neurons are activated by food-, morphine-, cocaine-, and alcohol-related stimuli [9,10,11,12,13]. Behavior that is motivated by drugs under high-demand conditions, and the motivation for drug-seeking that is potentiated by drug-conditioned stimuli, have been shown to involve the orexin system [14,15]. Nearly half of Orx neurons in rats express μ-opioid receptors, the main target of opioids [16,17]. Pharmacologically targeting the Orx system may therefore reduce opioid intake, craving, and relapse.

The present review discusses preclinical studies that demonstrated the involvement of the Orx system in substance use disorder in general and prescription OUD in particular.

## 2. The Orexin System and Substance Use Disorder

Orexin A (OrxA) and orexin B (OrxB), also called hypocretin 1 (Hcrt1) and hypocretin 2 (Hcrt2), respectively, are neuropeptides that were independently discovered in 1998 by two different research groups [18]. OrxA and OrxB derive from a common precursor (prepro-orexin) and are post-translational products of the *Orx* neuropeptide precursor gene. Orexin cell bodies are found in the lateral hypothalamus (LH) [19], the perifornical hypothalamus, the dorsomedial hypothalamus, and some scattered cells in the paraventricular nucleus [20]. Evidence indicates that reward-seeking functions are primarily associated with the recruitment of Orx cells in the LH [21,22], whereas arousal- and stress-related processes are associated with the recruitment of Orx neurons in the dorsomedial hypothalamus/perifornical hypothalamus [21,22]. Orexin receptor 1 (OrxR1; Hcrt-r1) and orexin receptor 2 (OrxR2; Hcrt-r2) [18,23,24] are involved in the regulation of highly motivated behavior, stress, and arousal [8,22,25,26,27,28,29,30,31], suggesting the potential of targeting them for the treatment of addiction. *OrxR1* and *OrxR2* mRNA expression is found throughout the brain [32,33], and each receptor subtype plays a different physiological role. The prefrontal cortex exhibits high *OrxR1* mRNA expression, and the nucleus accumbens expresses high *OrxR2* mRNA expression [32]. OrxR1 signaling has mainly been implicated in reward-seeking behavior, whereas OrxR2 signaling has been implicated in arousal and sleep/wake cycle regulation [22].

The orexin system has been shown to contribute to the regulation of motivation, especially highly motivated behavior, arousal, and stress, making this system an ideal target for addiction treatment [8,22,25,26,27,28,29,30,31]. The Orx system plays a prominent role in the motivation for reward (including drugs of abuse), stress, and anxiety [8,22,25,26,27,28,29,30,31], all of which contribute to problematic drug abuse [34,35]. Neural systems that are involved in processing natural rewards and drugs of abuse overlap; therefore, neuroplasticity that is caused by drug exposure may be responsible for maladaptive, compulsive, and addictive behavior [36,37,38]. Pharmacological manipulation of the Orx system is particularly effective in modifying the conditioned effects of drug cues in conditioned place preference and reinstatement studies [9,39,40]. We recently found that the Orx system is engaged to a greater extent by drugs of abuse than by natural non-drug reinforcers [1]. For example, although stimuli that were conditioned to cocaine, alcohol, and a conventional reinforcer (i.e., palatable sweet solution) were equally effective in eliciting reinstatement, the OrxR1 antagonist SB334867 selectively reversed the conditioned reinstatement that was induced by a cocaine- or alcohol-related stimulus, but had no effects on the same stimulus that was conditioned to a conventional reinforcer ([11,41,42] this laboratory). One possible explanation for the preferential reversal of conditioned reinstatement for drugs vs. non-drugs by SB334867 could be that the drug-induced neuroadaptation to neural systems that controls motivation under normal conditions (i.e., toward conventional reinforcers) is only revealed when blocking OrxR1 with SB334867. There is evidence of drug-induced dysregulation of Orx transmission. Earlier findings revealed that *Orx* mRNA expression is upregulated in the LH in inbred alcohol-preferring (iP) rats following chronic alcohol consumption [39]. Moreover, Orx production has been reported to be affected by opioids. Nine days after 1 week of 3 h/day heroin self-administration, a significant increase in *Orx* mRNA levels was observed in the LH [43]. Other studies have reported greater *Orx* expression during acute withdrawal from chronic morphine or heroin exposure [44,45]. Furthermore, it was recently reported that a higher number of Orx cells are found in the postmortem brains of heroin addicts [46], and in rodents exposed to long term morphine [46] or cocaine [47,48], suggesting that increased numbers of Orx-expressing neurons may have a crucial role in defining propensity and severity in the addiction phenotype. Because OrxA has been found to decrease the excitability of brain reward systems in the LH [49], it is possible that alterations of *Orx* expression may also be involved in negative affective states that are associated with opioid withdrawal and abstinence, which may be important in the modulation of opioid-seeking behavior [9,50].

One class of OrxR antagonists has been synthesized to target OrxR1. These single OrxR antagonists (SORAs), have 50-fold greater selectivity for OrxR1 over OrxR2 [24,51]. For example, *N*-(2-methyl-6-benzoxazolyl)-*N*’-1,5-naphthyridin-4-yl urea (SB334867) [52,53] has been shown to have very high selectivity for OrxR1, bioavailability, and potency [24,51]. SORAs have also been synthesized to specifically target OrxR2, with ≥ 250-fold selectivity for OrxR2 over OrxR1 [24,51], and the capacity to reduce numerous addiction-like behaviors. Findings that have described the use of SORAs that target OrxR2 on drug-motivated behavior are inconsistent vs. OrxR1-specific SORAs, such as SB334867. A third class of OrxR antagonists, referred to as dual OrxR antagonists (DORAs), target both OrxR1 and OrxR2, and have been developed to treat insomnia. Examples of DORAs include almorexant ([2*R*]-2-[(1*S*)-6,7-dimethoxy-1-(2-[4-(trifluoromethyl)phenyl]ethyl]-3,4-dihydro-1*H*-isoquinolin-2-yl)-*N*-methyl-2-phenylacetamide [ACT078573]) [54,55] and suvorexant ([(7*R*)-4-(5-chloro-1,3-benzoxazol-2-yl)-7-methyl-1,4-diazepan-1-yl]-[5-methyl-2-(triazol-2-yl)phenyl]methanone [MK4305]) [56]. To date, although most of the research in the field that has studied the effects of DORAs on addiction-like behaviors has tested almorexant [57,58,59], interest in the use of suvorexant [60,61] has recently arisen. Suvorexant was approved for the treatment of insomnia in the United States in 2014 (for review, see [62]), and could be used to treat drug-induced sleep disruptions (e.g., [63]) that are a significant contributor to relapse. Most preclinical studies that have examined the efficacy of blocking OrxRs tested only acute antagonist administration. Repeated OrxR1 inhibition, under some conditions, has been shown to exert longer-lasting preventive effects against drug-related, cue-induced cocaine-seeking [64]. The orexin neurons interface with several neural systems and are involved in various physiological processes. Repeated OrxR inhibition may cause nonspecific side effects by influencing numerous neural pathways. Dysfunctions of the Orx system can cause narcolepsy [65,66,67], while pharmacological manipulations of the Orx system can have nonspecific side effects on food intake [68,69,70], inhibitory responses [71,72,73], and learning and memory [74,75,76]. Such untoward effects need to be considered when investigating the clinical potential of OrxR antagonist treatment.

## 3. Manipulation of the Orexin System to Treat Prescription Opioid Use Disorder

The interest in testing OrxR antagonists for the treatment of prescription OUD was spawned by earlier studies that suggested that the Orx system has a function in regulating the high motivation to take and seek drugs of abuse [77,78,79,80,81,82]. Some recent studies have investigated the potential of targeting the orexin system for the treatment of prescription opioid misuse. OrxR1 antagonism was shown to effectively reduce the motivation for oxycodone, fentanyl, and remifentanil in preclinical rodent studies [83,84,85,86,87].

We recently reported that rats self-administered oxycodone under a long-access schedule. During these 12 h daily sessions, the rats exhibited a significant increase in oxycodone intake and signs of oxycodone dependence [83]. Discriminative stimuli (S^D^) were conditioned to oxycodone self-administration and eventually gained the ability to reinstate oxycodone-seeking, and this conditioned effect was resistant to extinction over five presentations of the S^D^ (i.e., 30 days after the last oxycodone self-administration session). Importantly, this study also found that OrxR1 blockade with SB334867, but not OrxR2 blockade with TCSOX229, decreased oxycodone intake (Table 1), which was consistent with studies of heroin, fentanyl, and remifentanil self-administration [84,85,87,88,89]. However, these oxycodone data partially contrasted with findings on cocaine self-administration, in which OrxR1 blockade with SB334867 did not affect cocaine self-administration [77,90], unless the contingency to take cocaine was kept under a high-effort schedule (e.g., high fixed-ratio or progressive-ratio schedule of reinforcement; for a review, see [91]). Notably, however, *Orx* silencing reduced cocaine self-administration under a low-effort schedule (i.e., FR1 timeout 20 s schedule of reinforcement) [92] under extended-access conditions (6 h/day). These findings suggest the feasibility of normalizing compulsive drug intake in addicted individuals by inhibiting Orx signaling. We also found that the OrxR1 antagonist SB334867, but not the OrxR2 antagonist TCSOX229, prevented the S^D^-induced conditioned reinstatement of oxycodone-seeking behavior ([83], Table 1), thus supporting the involvement of Orx, particularly in reward-seeking that was elicited by external stimuli when the motivation for drug-seeking was augmented by giving stimuli that were conditioned to the drug [15]. Requiring further exploration are the mechanisms by which SB334867 produced alterations of oxycodone-seeking and -taking behavior in our study [83]. SB334867 may lower the general motivation for oxycodone, reflected by decreases in self-administration and reinstatement, suggesting the involvement of OrxR1 in oxycodone’s reinforcing effects and oxycodone-related, S^D^-driven, appetitive behavior. Numerous studies reported that cue- and context-induced reward-seeking behavior for cocaine, heroin, alcohol, fentanyl, and sucrose involved OrxR1-mediated Orx transmission [12,28,39,40,42,87,88,93,94]. Overall, these results demonstrate that OrxR1 is implicated in the mediation of the reinforcing effects of oxycodone and appetitive behavior that is controlled by oxycodone-related stimuli, and may be a promising therapeutic target for the treatment and prevention of prescription OUD.

Rats will also voluntarily self-administer remifentanil, a µ-opioid agonist with a short duration of action, and achieve similar breakpoints to heroin on a progressive-ratio schedule of reinforcement [95]. In 2015, Porter-Stransky et al. [84] employed a behavioral economics approach in rats and observed extensive individual variability in the intake of the ultra-short-acting opioid remifentanil [84]. In a behavioral economics procedure, the Q_0_ value is a hypothetical measure of the amount of drug an individual would use while the drug is entirely free (i.e., free consumption, likewise a measure of the hedonic set point). The Q_0_ value is measured by a demand curve intercept of the ordinate and the slope of the demand curve. The demand elasticity, α, denotes how consumption fluctuates relative to cost independently of Q_0_ [96,97]. These behavioral economics demand curve approaches are useful for measuring the motivation for drugs, because they capture individual differences in the motivation for drugs, enable comparisons between different drugs, and offer unique parameters of motivation. Briefly, rats with a low baseline Q_0_ (i.e., low takers) exhibit high demand elasticity, α (i.e., lower responding as the drug price increases, signifying low motivation for the drug), while rats with a high Q_0_ (i.e., high takers) show low demand elasticity, α, by continuing to self-administer the drug in spite of the higher cost, thus reflecting higher motivation for the drug. Using this model, the authors found that the OrxR1 antagonist SB334867 reduced the motivation to self-administer remifentanil only in low-taker rats (Table 2). Similarly, SB334867 attenuated the cue-induced, but not drug-induced, reinstatement of remifentanil-seeking only in the low-taker group (Table 2). These results appear to be in contrast with previous observations of the effects of OrxR1 blockade on the motivation for different drugs of abuse (e.g., cocaine and alcohol). Indeed, SB334867 was shown to more effectively reduce alcohol drinking in high-alcohol-preferring rats [98] as well as intake- and cue-induced reinstatement in rats with a high demand for cocaine [99,100]. These differences support the hypothesis that different neurobiological mechanisms mediate the motivation for different drugs of abuse. Two follow-up studies from the same group used the behavioral economics approach, and confirmed the ability of SB334867 to reduce the motivation for remifentanil and decrease cue-induced reinstatement ([85,86], Table 2). Moreover, they identified the ventral pallidum as an important brain region for the regulation of the motivation for remifentanil and cue-induced reinstatement [85,86]. The ventral pallidum receives Orx inputs, has high *OrxR1* mRNA expression [20,32], and has been critically linked to the drive for reward-seeking behavior [101,102,103,104], suggesting that this region may be a site where Orx modulates reward behavior. Interestingly, the authors showed that the inhibition of remifentanil-seeking by SB334867 persisted beyond the pharmacological availability of SB334867 [86].

Another recent study investigated the efficacy of SB334867 in reducing the motivation for fentanyl ([87], Table 3). Using the behavioral economics procedure, the study found that rats readily self-administered fentanyl and exhibited somatic signs of withdrawal. The level of motivation for fentanyl predicted the magnitude of the reinstatement of fentanyl-seeking behavior that was induced by the presentation of stimuli that were previously concomitant with the drug. Further corroborating the potential use of SB334867 for the treatment of prescription OUD, the study also showed that systemic pretreatment with SB334867 decreased the motivation for fentanyl without altering sucrose self-administration. The blockade of OrxR1 with SB334867 reduced the cue-induced reinstatement of fentanyl. Orexin neurons are activated in response to drug-associated cues and contexts [13,89,105], and OrxR1 functionality is crucial for the cue-induced reinstatement of oxycodone-, heroin-, remifentanil-, cocaine-, alcohol-, nicotine-, and sucrose-seeking [83,84,88,93,105,106]. Importantly, the authors of this study [87] found a positive association between the motivation for fentanyl and the efficacy of SB334867 in reducing fentanyl intake and seeking, underscoring the relevance of OrxR1 signaling to the maintenance of motivated behavior, especially in individuals that exhibit high motivation for the drug. Although Orx neurons have been shown to be activated by food-related cues [9,107], SB334867 can selectively decrease drug-seeking without interfering with regular food intake in ad libitum-fed animals [77,108,109], or reward-seeking that is produced by stimuli that are conditioned to a palatable sweet solution [42,94]. Such findings demonstrate that OrxR1 may be a viable treatment target to selectively reduce the craving and motivation for prescription opioids. One common limitation of these studies, however, is that the efficacy of an OrxR2 antagonist was not tested [84,85,86,87], in contrast to our study with oxycodone [83]. Thus, we cannot completely exclude the possibility that OrxR2 signaling may also regulate the motivation for remifentanil and fentanyl. OrxR2 signaling mediates stressful states and arousal, aspects that characterize substance use disorders in general. In fact, a recent study showed that antagonizing OrxR2 with NBI-80713 produced a significant decrease in heroin self-administration only in rats that were made heroin-dependent [110], strongly suggesting that OrxR2 might play an important role in prescription OUD. Future studies are warranted to systematically explore the role of OrxR2 signaling, both alone and combined with OrxR1 signaling, to better understand the potential to target the orexin system to treat prescription OUD.

## 4. Potential Use of DORAs to Maintain Abstinence and Prevent Relapse

Acute treatment with opioids for pain management appears to improve sleep quality [111]. However, as use continues, sleep deficits can occur [112]. Sleep architecture is altered as opioid dependence and withdrawal develop [113,114]. Alterations of sleep architecture that occur during withdrawal from short-term opioid administration may be different from changes that occur during withdrawal from chronic use. Significant insomnia commonly occurs among opioid users long after withdrawal, characterized by increases in sleep latency and time awake after sleep onset, and accompanying frequent arousals [115]. Withdrawal-associated sleep problems, including sleep loss and worse sleep quality, have been suggested to contribute to opioid use and relapse. Sleep disturbances are prominent symptoms of substance-related disorders and could account for a substantial proportion of sleep problems [63]. Opioid exposure is known to directly suppress the hypothalamic Orx system [116], perhaps explaining the lethargy and cognitive dysfunction that are induced by opioid use [117,118]. Shortly after its discovery, the key role of the Orx system in wakefulness became clear, raising the possibility that OrxR antagonism may be a new therapeutic approach for the treatment of insomnia by blocking the Orx-mediated drive of wakefulness [54,119]. Under physiological conditions, Orx neuron activity and peptide levels show diurnal variations. High Orx activity is correlated with wakefulness, and low Orx activity is correlated with sleep [120,121,122]. The wake-promoting functions of Orx are supported by the finding that arousal is increased by the administration of exogenous Orx peptides and the optogenetic activation of Orx neurons [119,123,124,125]. Nevertheless, the neurobiology of sleep/wake cycling is complex and regulated by interactions between sleep-promoting systems (e.g., the inhibitory γ-aminobutyric acid system) and wake-promoting systems (e.g., Orx, acetylcholine, and monoamines) [126,127]. 

Consistent with the sleep-promoting role of Orx, SORAs that are selective for OrxR2 were shown to have sleep-promoting properties, whereas SORAs that are selective for OrxR1 were largely ineffective with regard to promoting sleep [128,129]. In addition to SORAs, some DORAs have been synthesized as potential therapeutics for the management of sleep/wake disorders [62]. Interestingly, OrxR2 SORAs appear to require a higher receptor occupancy to achieve their effect on sleep maintenance compared with DORAs, suggesting that OrxR1 also plays a role in sleep maintenance [128]. DORAs block the activity of OrxR1 and OrxR2 to reduce the threshold of the transition to sleep and attenuate Orx-mediated arousal [62]. Among DORAs, suvorexant has been approved by the United States Food and Drug Administration for the treatment of insomnia (for a review, see [62]). Because of the efficacy of suvorexant in treating sleep disorders and the efficacy of OrxR antagonists in reducing “drug motivation,” some clinical trials are now studying the efficacy of suvorexant in normalizing sleep, craving, and stress states in patients who are affected by substance use disorders, including OUD [130,131]. However, even though clinical trials are presently ongoing to examine the efficacy of suvorexant on OUD (see [130,131]), to our knowledge no data are available yet. Moreover, other potential OrxR antagonists have been synthesized for the management of sleep disorders [62,132]. One major obstacle to the treatment of OUD is the recurrence of relapse to opioid misuse even after a long time of imposed or voluntary abstinence [2]. Chronic opioid use induces the development of physical dependence and withdrawal symptoms when drug use is interrupted. Withdrawal symptoms gradually fade within a few days but can sometimes last for weeks after use is discontinued [2]. In patients who suffer from prescription OUD, insomnia is a major withdrawal symptom that might ultimately represent one of the major factors that triggers relapse. Thus, alleviating sleep disturbances during opioid withdrawal might attenuate relapse vulnerability. We hypothesize that the sleep-promoting Orx system is compromised as opioid dependence and withdrawal develop. It has been shown that suvorexant exhibits a long half-life (up to 13 h), and depending on the dose administered, sufficient occupancy of OrxRs occurs [132,133]. Therefore, daily treatment with a DORA compound, such as suvorexant, in the evening within 30 minutes of bedtime, should not only be sufficient to relieve sleep disturbances during opioid withdrawal but could also reduce craving and attenuate relapse vulnerability the following day [130,132]. 

## 5. Conclusions

To date, only a few studies have investigated the efficacy of SORAs in reducing the intake and reinstatement of synthetic prescription opioids, and most of them were focused on OrxR1, neglecting the potential importance of OrxR2. Moreover, no preclinical study has reported the use of DORAs to treat prescription OUD, indicating that more preclinical work would be needed to elucidate the ways these compounds can be beneficial in the clinical conditions of prescription OUD. To achieve and maintain abstinence, treatments for prescription OUD should alleviate not only symptoms of craving and the motivation for the drug, but they should also reduce anxiety-related states provoked by opioid abuse and sleep disturbances that occur as dependence and withdrawal develop. With regard to the anxiety-related states induced by opioids, clinical studies have demonstrated the participation of the Orx system in the emotive perturbation that occurs during alcohol withdrawal [134,135]. This could also be the case for prescription opioids. Knowing the extensive projections of the Orx system throughout the brain, its role is not likely to be limited to sleep regulation, but encompasses other neuropsychiatric disorders, such as anxiety, depression, and prescription OUD. As of today, only a limited amount of preclinical studies suggest a role of OrxRs in prescription OUD, and even though more investigations are needed to elucidate the ways in which prescription opioids perturb the Orx system as dependence and withdrawal develop, the existing literature strongly supports the hypothesis that the inhibition of Orx signaling could be an original approach for the treatment of prescription OUD.

## Figures and Tables

**Table 1:** Summary of the effect of OrxR blockade on oxycodone-related behaviors.

OXYCODONE	Procedure	Species	Sex	SORAs OrxR1	SORAs OrxR2	DORAs
**Intake**	**self-administration**	**rat**	**male**	**↓**	**–**	nt
**Reinstatement**	**cue-induced**	**rat**	**male**	**↓**	**–**	nt

Data from [83]; ↓ = decrease; **–** = no effect; nt = not tested.

**Table 2:** Summary of the effect of OrxR blockade on remifentanil-related behaviors.

REMIFENTANIL	Procedure	Species	Sex	SORAs OrxR1	SORAs OrxR2	DORAs
**Intake**	**self-administration**	**rat**	**male**	**↓**	**nt**	**nt**
**Reinstatement**	**cue-induced**	**rat**	**male**	**↓**	**nt**	**nt**
	**drug-induced**	**rat**	**male**	**–**	**nt**	**nt**

Data from [86,87,88]; ↓ = decrease; **–** = no effect; nt = not tested.

**Table 3:** Summary of the effect of OrxR blockade on fentanyl-related behaviors.

FENTANYL	Procedure	Species	Sex	SORAs OrxR1	SORAs OrxR2	DORAs
**Intake**	**self-administration**	**rat**	**male**	**↓**	**nt**	**nt**
**Reinstatement**	**cue-induced**	**rat**	**male**	**↓**	**nt**	**nt**

Data from [87]; ↓ = decrease; nt = not tested.

## Abbreviations

Orx	orexin
OrxR1	orexin receptor 1
OrxR2	orexin receptor 2
SORAs	single orexin receptor antagonists
DORAs	dual orexin receptor antagonists
SB334867	*N*-(2-methyl-6-benzoxazolyl)-*N*’-1,5-naphthyridin-4-yl urea
